# Antibiotic Resistance Genes in Global Food Transformation System: Edible Insects vs. Livestock

**DOI:** 10.3390/foods13203257

**Published:** 2024-10-13

**Authors:** Rifat Nowshin Raka, Lin Zhang, Rui Chen, Xiaofeng Xue

**Affiliations:** State Key Laboratory of Resource Insects, Institute of Apicultural Research, Chinese Academy of Agricultural Sciences, Beijing 100093, China; rnraka92@gmail.com (R.N.R.);

**Keywords:** antibiotic-resistant genes (ARGs), entomophagy, farm animals, sustainable food systems

## Abstract

Antibiotic-resistant genes (ARGs) pose a significant threat to the global food transformation system. The increasing prevalence of ARGs in food has elicited apprehension about public health safety. The widespread dissemination of ARGs in food products, driven by the inappropriate use of antibiotics, presents significant adversity for the safety of emerging future food sources such as edible insects. As the world faces increasing challenges related to food security, climate change, and antibiotic resistance, edible insects offer a sustainable and resilient food source. The intriguing possibility of edible insects serving as a less conducive environment for ARGs compared to livestock warrants further exploration and investigation. In this recent work, we listed ARGs from edible insects detected so far by in vitro approaches and aimed to construct a fair comparison with ARGs from livestock based on relevant genes. We also presented our argument by analyzing the factors that might be responsible for ARG abundance in livestock vs. edible insects. Livestock and edible insects have diverse gut microbiota, and their diets differ with antibiotics. Consequently, their ARG abundance may vary as well. In addition, processed edible insects have lower levels of ARGs than raw ones. We hypothesize that edible insects could potentially contain a lower abundance of ARGs and exhibit a diminished ability to disseminate ARGs relative to livestock. A regulatory framework could help intercept the increasing prevalence of ARGs. Due diligence should also be taken when marketing edible insects for consumption.

## 1. Introduction

Antibiotic resistance (AR) has been defined as the “quintessential” health concern. Due to the overuse of antibiotics in agriculture, the food industry, and pharmaceuticals, the issue is growing serious day by day, leading to an increase in drug-resistant infections in humans and animals. The antibiotic-resistant genes (ARGs) responsible for causing these infections are prevalent and diverse in environments where AR selection and transmission occur regularly. ARGs can spread through bacteria in livestock, dairy, shellfish, and other food sources. They reach animals via surface, groundwater, soil, and air [1]. AR poses a significant threat to public health, resulting in approximately 4.95 million deaths worldwide and 1.3 million fatalities annually in China alone [2,3]. Due to the lack of new antibiotics development, it is crucial to practice antibiotic stewardship immediately.

Foods are quite vulnerable to ARG propagation. Novel food substitutes like edible insects are not out of this spectrum. Edible insects are emerging as a promising global food transformation system component. Their high nutritional value and relatively low environmental impact make them a compelling alternative to traditional livestock. According to nutritional data, edible insects are a promising source of micronutrients (copper, zinc, manganese, phosphorus, selenium, riboflavin, pantothenic acid, biotin, and folic acid) as well as proteins [2,3,4]. Furthermore, research has demonstrated that edible insect fiber, chitin, and omega-3 fatty acids are beneficial for both human and animal gut bacteria [5]. Insect-based food has a long history in Southeast Asia, and insects like crickets, grasshoppers, and mealworms are commonly consumed in tropical and subtropical regions. While insects are not yet widely consumed in other parts of the world, their acceptance and integration into traditional cuisines are on the rise [4,6]. Out of 2000 edible insect species, the European Union has authorized four so far in frozen, dried, and powdered form: *Tenebrio molitor* larvae, *Locusta migratoria*, *Acheta domesticus*, and *Alphitobius diaperinus*. By incorporating insects into our diets, we can diversify our food systems, reduce our reliance on resource-intensive animal agriculture, and contribute to a more sustainable future. Therefore, determining the risk factor for edible insects is becoming crucial. The risk of using insects as food ingredients or in food product manufacturing is unclear [7]. In terms of microbiological risks, insects can carry bacteria species in their gut and cuticle. They can also be a source of transferable ARGs that are associated with edible insects and opportunistic human pathogens [8,9]. However, while the use of antibiotics in livestock is a major contributor to ARGs, edible insects typically use fewer antibiotics, reducing the likelihood of breeding resistant strains [10,11]. Moreover, the comparative simplicity of insect microbiomes compared to those of farm animals may impede the transfer of antimicrobial resistance genes [12,13].

We hypothesized that the abundance of ARGs in insects could be lower than in animals because of the differences in their diet, microbial diversity, and farming process. Given the frequency and transmission of ARGs, edible insects may be less threatening than animals. Additionally, appropriate processing may assist in mitigating ARGs by minimizing the microbial load in edible insects.

## 2. Prevalent ARGs in Insects and Livestock

**Figure 1:**
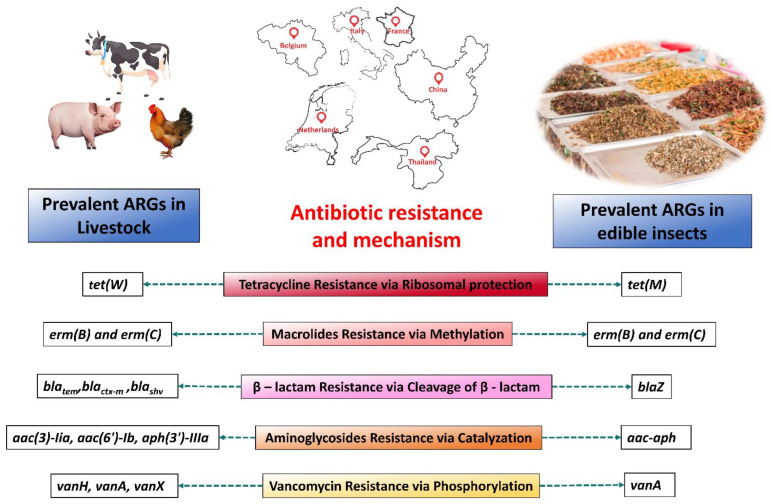
Comparison of prevalent ARGs in Dutch, Belgian, Italian, Chinese, Thai, and French edible insects and livestock based on 5 major antibiotic-resistant genes according to the available literature. Color shades change based on the frequency, where the red shade has the highest frequency and the yellow shade has a comparatively low frequency of ARG occurrence. Locations are based on our compared evidential literature.

Only a handful of studies revealed a widespread prevalence of genes in edible insects that confer resistance to different antibiotics. Nonetheless, the extent of human exposure to ARGs carried by market-available edible insects is far from being determined, let alone the wild-reared ones (Table 1). ARGs in livestock, however, have been the subject of substantial research. When comparing the prevalence of ARGs between edible insects and livestock, a complex narrative emerges, highlighting the diverse microbial landscapes in these food sources (Figure 1).

While both edible insects and livestock harbor ARGs, their distribution and prevalence vary significantly. For instance, edible insects exhibit a notable prevalence of *tet* genes, while livestock demonstrates a higher abundance of *tet* and *erm* genes, along with several *bla* gene strains. Addressing AR in food sources requires a holistic understanding of microbial ecology and targeted interventions to promote sustainable farming practices and safeguard public health.

Edible insects exhibit a notable prevalence of certain ARGs, such as *tet(K)*, *tet(M)*, and *tet(S).* Studies by Milanovic and colleagues underscore the widespread occurrence of these genes in edible insects, with high positivity rates observed in samples collected from Thailand and the Netherlands. *Tet(K)* demonstrated the highest prevalence, with 90.9% positivity among 11 samples from those regions, followed by *tet(S)* with 54.5% positivity [8]. Similarly, ready-to-eat grasshoppers and mealworms from various regions of Europe displayed a high distribution of *tet(M)*, *tet(K)*, and *tet(S)*, indicating a consistent pattern across different insect species and geographical locations [17,19]. Conversely, livestock, particularly in Europe and selected Asian regions, demonstrate a higher prevalence of genes like *tet(W)*, *tet(A)*, *tet(B)*, and *tet(M)*. Research by Yang et al. and Jalencu elucidates the dominance of these genes in pig and duck farms, emphasizing their widespread presence within conventional livestock production systems [21,22].

In addition to the *tet* genes, the prevalence of *erm(B)* and *erm(C)* in both edible insects and livestock is worth noting. While these genes are less frequent in edible insects compared to *tet* genes, they still exhibit a significant presence, particularly in European insect species and livestock populations. For example, Milanovic et al., in 2016, found 45.4% and 18.2% of edible insect samples from the Netherlands and Thailand were positive for *erm(B)* and *erm(C),* respectively [8]. In 2017, Osimani and his team found consistent prevalence for *erm(B)* and *erm (C)* in ready-to-eat grasshoppers from the Netherlands, Thailand, and Belgium [23]. *Erm(B)* was the most abundant (57.5%) in France and Thailand, while no positivity was found in Belgium and the Netherlands [16]. *Erm(B)* is the most abundant in European livestock, followed by *Erm(C).* In several animal farms in northern and southern China, *erm(B)* and *erm(C)* were found to be abundant [24,25]. In a study of pig farms in 25 Thai provinces, *Erm(B)* was also found to be resistant to primary microlide [26]. The consistency of these findings across different studies underscores the importance of understanding the broader microbial ecology of food sources to mitigate the spread of antibiotic resistance.

However, it is essential to acknowledge that the prevalence of the *bla* gene strains appeared somewhat higher in livestock than in edible insects. Studies by Milanovic et al. and Osimani et al. reveal a comparatively lower occurrence of *blaZ* and *mecA* in edible insects from Europe and Thailand, suggesting potential differences in the mechanisms of antibiotic resistance between both protein sources [8,15,20]. Furthermore, the prevalence of extended-spectrum β-lactamase (ESBL) genes, e.g., *bla_tem_ bla_ctx-m_*, and *bla_shv_* in livestock, particularly in chicken meat, highlights the need for targeted interventions to address antibiotic resistance in conventional livestock farming [27].

Although vancomycin resistance and aminoglycosides are rarely seen in livestock or edible insects, recent research by Osimani et al. shows that ready-to-eat mealworms and grasshoppers from the European market have a low frequency of *aac-aph* [17,23]. Vancomycin-resistant genes *vanA* and *vanB* have also been reported occasionally in both dietary sources. *Aac(3)-Iia*, *aac(6′)-Ib*, and *aph(3′)-IIIa* have all been found in cattle, chicken, and swine in Europe, South Korea, and China, even though utilizing aminoglycosides in livestock is discouraged due to potentially dangerous residues [28,29,30,31]. It is interesting to note that *van(A)* is the most common vancomycin-resistant gene in cattle; however, it is not that common compared to other ARGs.

This comparative analysis of ARG prevalence in edible insects and livestock reveals a nuanced landscape, with distinct patterns emerging across different gene families and animal sources.

Unfortunately, there is a huge gap in research regarding ARGs in edible insects. Studies are restricted to PCR methods with gut-specific samples of edible insects, whereas studies on livestock have concentrated on metagenomics and waste samples. It remains unavoidable, however, that ARGs might be more common in livestock than edible insects.

## 3. Probable Reasons for ARG Abundance: Edible Insects vs. Livestock

Addressing AR in food sources requires a holistic understanding of microbial ecology and targeted interventions to promote sustainable farming practices and safeguard public health. The gut microbiome is the primary determinant of the abundance of ARGs in edible insects and animals. The consumption of antibiotics and diets are important factors as well. Environmental factors like pollution and waste production can also contribute to the abundance of ARGs in edible insects and livestock.

### 3.1. Gut Microbiome

Insects can support a diverse autochthonous microbial community of viruses, fungi, bacteria, archaea, and protozoa. The intrinsic microbiota differs across different phases of development in insects and is linked to the gastrointestinal tract, cuticle, and other anatomical compartments. According to the scientific papers reviewed, fresh edible insects generally contain significant microbial loads. The main microbial concerns of edible insects include high total counts of mesophilic aerobic bacteria (3.6–9.4 log cfu/g) and the presence of spore-forming bacteria (0.5–5.8 log cfu/g) that can withstand heat treatment; for example, *Enterococcaceae* (4.2–7.8 log cfu/g), *Staphylococcaceae*, yeast and molds (3.4–7.2 log cfu/g), lactic acid bacteria (5.2–9.1 log cfu/g), psychrotrophic aerobes (4.5–7.2 log cfu/g), and *Enterobacteriaceae* (3 log cfu/g threshold) [32]. Insects may also carry food spoilage, harmful microorganisms, and perhaps pathogenic species [33]. However, all the genera are involved in specific effects on the food safety of edible insects, making them indicators of hygiene. The global microbial load of a food sample can be ascertained by counting all mesophilic aerobes; this will also reveal the sample’s overall contamination and microbiological quality. They can also be used to forecast the shelf-life of products held at room temperature. The presence of *Enterobacteriaceae* in the microbiota of edible insects suggests that they were not degutted or handled under adequate sanitary settings. Usually found in soil and dust, spore-forming bacteria can develop endospores that are difficult to eradicate due to their resistance to heat, dehydration, radiation, and chemicals. Since certain species of lactic acid bacteria (LAB) generate compounds that are good for human health or have probiotic potential, they are typically regarded as healthy, though some species can be poisonous. Psychrotrophic bacteria can cause food spoiling and should be considered while storing edible insects at low temperatures. Interestingly, properly processed edible insects have a trace amount of these microbiota. Boiling, sun-drying, and frying decrease the microbial counts in grasshoppers [19,23]. *Enterobacteriaceae* in fresh mealworms and small and large crickets can be reduced by boiling, roasting, and stir-frying [16,17,18]. Degutting is another way to avoid microbial contamination.

**Figure 2:**
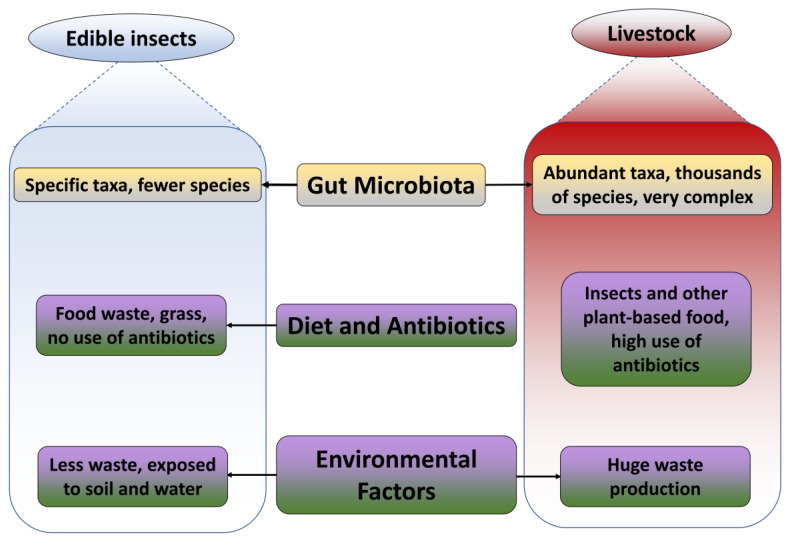
Comparison based on the reason for ARGs’ abundance in edible insects and livestock.

On the contrary, livestock have complicated microbial compositions with a variety of species numbers. Cattle, pig, sheep, and chicken guts contain a huge diversity of microbiota, the majority portion of which are *bacteriocides* and *firmicutes,* followed by *actinobacter*. The majority of gut microbiome studies in livestock have focused on the characterization of microbial communities using 16S rRNA gene amplicon sequencing as a result of the differences in animal diet composition, gastrointestinal tract (GIT) location, feed efficiency, breed specificity, metabolic disturbances, changes over time, and individual specificities, as well as across housing types and farms. A total of 80–90% of livestock microbiota is composed of *bacteriocides* and *firmicutes*. Other taxa include *actinobacteria*, *proteobacteria*, *spirochaetes*, *tenericutes*, *verrucomicrobia*, *fibrobacteres*, etc., differing in abundance between the host species [34,35]. Livestock contains thousands of species, including opportunistic and harmful microbes. As a result, ARGs become more prevalent and resistant to a wide range of antibiotics. For instance, 30 ARGs correlated with colistin and daptomycin were detected in the rumen of sheep [36]. Non-ruminant livestock species face an even more difficult predicament. Major ARGs in hens are similar to those found in pigs, including tetracycline, aminoglycoside, and macrolide–lincosamide–streptogramin [37] (Figure 2).

Vertical transmission is the term for the process by which ARGs can multiply through genetic transfer within the same species of these bacteria. Resistance genes allow a species to adapt and live in circumstances where antibiotics are present. This happens when these genes are passed down from one generation to the next. Since the intestinal ecosystem of edible insects is less diverse than that of animals, there is likely less frequency of this genetic transfer.

### 3.2. Diet and Antibiotics

Diet is the main culprit for the potential risk factors in the food source. The main diet for edible insects is plant-based substrates like grains or dry vegetables. They eat things we normally do not eat, like apple cores and vegetable waste. Insect diets differ (for example, grasshoppers feed on grass, but *Tenebrio molitor* grows primarily on grain matrices). While livestock also feed on plant-based diets such as corn, soybeans, sorghum, oats, and barley, there is a plethora of research for improved and composed diets. However, antibiotics are often used as growth promoters to acquire more meat and to encourage livestock’s rapid growth [38]. In the case of insects, there is seldom a need for antibiotics and growth promoters. As regular exposure to antibiotics for the long term is one of the main reasons for ARG abundance, edible insects can easily dodge this bullet.

### 3.3. Environmental Factors

Horizontal transfer, pollution, and waste production are environmental factors worth mentioning. ARGs are largely dispersed by horizontal gene transfer from the environment. Bacteria can use processes, including transformation, transduction, and conjugation, to pick up resistance genes from their environment, such as soil or water. As a result, bacteria acquire novel resistance features that increase their chances of surviving in settings where antibiotics are abundant. Antibiotics are not the only substances that select ARGs; therefore, this co-occurrence is significant. ARG growth may also be aided by environmental heavy metal pressure, which co-selects antibiotic and metal resistance. Studies reveal that selection for antibiotic resistance is also driven by the co-selection of genes resistant to other toxic substances, including disinfectants, nanomaterials, and biocides [39,40,41]. The amount of ARGs in bacteria containing aromatic degradation genes (ADGs) is more than double that of bacteria without ADGs [42]. P-nitrophenol, p-aminophenol, and phenol are examples of phenolic compounds that have been shown in a study to dramatically raise the frequency of ARG horizontal transfer [43].

Livestock produce a large amount of excrement and are visibly high in ARGs, which can contaminate land, water, and air. Conventional livestock waste treatment does not completely remove antibiotic-resistant bacteria (ARBs), causing their release into soil and water environments [44]. Research shows swine and chicken farms have higher ARG concentrations than hospital and municipal wastewaters, possibly due to higher levels of residual antibiotics from consistent antibiotic use [45]. Edible insects produce little or no waste because they are consumed whole. Moreover, insect farming is a sustainable approach that involves feeding insects with agricultural waste, such as unwanted plant stems and food scraps, in order to promote circular economies. These insects could consume food and biomass that would otherwise go to waste, promoting resource recycling and reuse. They may carry a significant number of bacteria on the surface and within their bodies, which can only be safely consumed if properly treated and kept [10]. Antimicrobial peptides are easily detected in edible insects, specific in types, and have already been linked to significant health benefits [46].

Conclusively, edible insects may offer a more sustainable and safer protein source compared to conventional livestock due to their lower prevalence of ARGs, reduced antibiotic usage in production, and minimal environmental impact. Edible insects benefit from a less diverse gut microbiome, reduced antibiotic exposure, and a more sustainable farming model, suggesting a potentially lower risk of ARG dissemination.

## 4. Pertinence of Edible Insects over Livestock: Our Perspective

The global food transformation system is undergoing a paradigm shift as we grapple with pressing challenges such as climate change, food insecurity, and the emergence of ARGs. The unsustainable practices of traditional livestock farming, coupled with the growing demand for protein, have created a pressing need for innovative and sustainable food sources. Edible insects, once considered a niche culinary curiosity, are emerging as promising solutions. Their nutritional value, environmental benefits, and potential to mitigate the threat of antibiotic resistance make them a compelling addition to our dietary repertoire.

**Figure 3:**
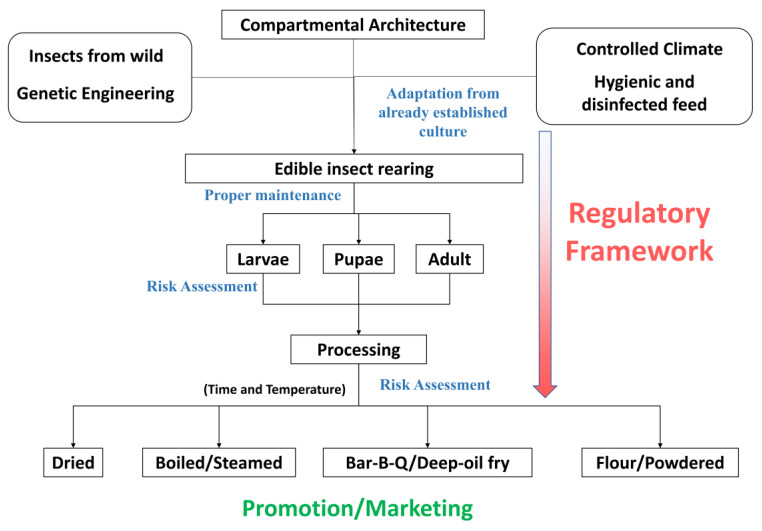
Proposed sequence for food safety regarding edible insects.

One of the most pressing concerns in the global food system is the increasing prevalence of ARGs, driven by the overuse of antibiotics in agriculture and medicine. Edible insects, which typically require fewer antibiotics compared to livestock, offer a potential solution to this problem. Do edible insects contain more ARGs than livestock? Our hypothesis is edible insects have a higher possibility of being safer than livestock in the context of ARGs. The basis of this concept is the simple and changeable gut microbiome of edible insects, as well as their extensive AMP production. The abundance of ARGs can occur in three ways: it is transferred genetically among the same species, horizontal transfer from the environment, and long-term exposure to antibiotics. The advantage of edible insects is that their food safety assessment can be maintained due to their easy farming process and their simple structure and gut microbiota composition (Figure 3).

**Figure 4:**
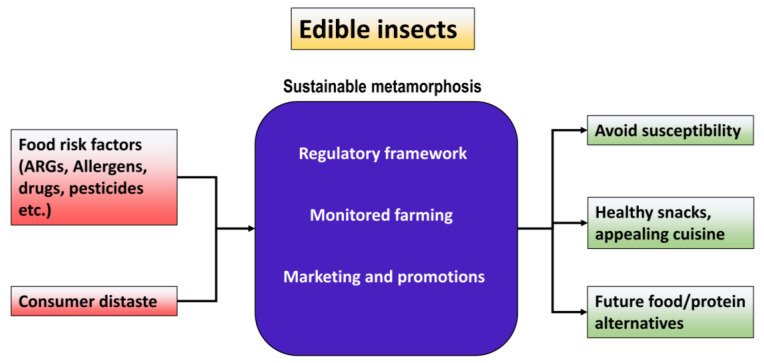
Sustainable metamorphosis for establishing edible insect convenience.

The first thing to be addressed is the safe treatment of rearing feed substrate. Ensuring contamination-free and hygienic feed is critical to healthy insect production. This is to ensure that “disinfecting the feed “and maintaining a “closed environment” for rearing are the best approaches. A closed environment can be maintained using “compartmental architecture” with climate-controlled rearing. As edible insects mature from larvae to adults, their gut microbial diversity varies. Therefore, certain bacteria are shredded, limiting the accumulation of ARGs. Genetic breeding and engineering can aid in this natural phenomenon of acquiring risk-free edible insects to rear industrially. This results in the establishment of a regulatory framework for edible insect cultivation. “Edible insects” must be considered “animal food” instead of “filth”, and risk assessment should be placed in every step from rearing to packaging. Nevertheless, insect farmers can adhere to livestock rules with few alterations. The actual challenge is to see if the regulations can be generalized for all edible insect species. Another important fact is that the processing methods for edible insects can reduce the microbiological risk to close to zero. Evidence showed that properly processed edible insects (dried, boiled, and powdered) had an ignorable presence of ARGs [18,47]. Last but not least, edible insects must be appealingly promoted as food. Every region can raise the insects they have in large quantities, but without consumer appeal, the industry will not establish itself successfully (Figure 4).

In regards to ARGs in edible insects, there is undoubtedly an enormous investigation dearth. Most of the research is still focused on the nutritional value of edible insects. ARGs in livestock have been studied more extensively, but they still lack a regional viewpoint. As we endeavor to comprehend the intricate workings of food production and antibiotic resistance in both food sources, future research holds immense potential to shed light on key aspects of this issue. Future research could focus on the prevalence and transmission dynamics of ARGs in edible insects and livestock, examining the mechanisms driving their dissemination and the impact of different farming practices on antibiotic resistance. To find the safe frequency of ARGs and provide a comparison, ARGs for edible insects and livestock should be calculated based on a sample per unit. For example, examining 1 kg of beef and 1 kg of *T. molitor* larvae simultaneously, either in their raw or processed forms. An extensive metagenomic study with more parameters and regional comparisons will be a key stepping stone. Identifying sustainable farming methods that minimize antibiotic use while promoting animal health and welfare can lead to a more resilient and environmentally friendly food system. Advancements in molecular biology and genomics offer opportunities for precision monitoring and surveillance of ARGs in both species. Resolving antibiotic resistance in food production systems requires multidisciplinary research and the incorporation of knowledge from ecology, public health, agriculture, and microbiology. Significant improvements and the implementation of evidence-based solutions require the cooperation of researchers, politicians, industry stakeholders, and consumers. As we strive to create a more sustainable and resilient food system, edible insects can unlock the potential to shape a healthier and more sustainable future. As awareness of their nutritional benefits and environmental advantages grows, cultural acceptance is likely to increase. Advancements in food technology and processing techniques will make edible insects more palatable and accessible to a wider audience.

## Figures and Tables

**Table 1:** Occurrence of ARGs in edible insects.

Insect	Scientific Name	Country	Bacteria	Methods	ARGs	Antibiotics	Ref.
Small crickets	*Acheta domesticus*	Netherlands Market	*Streptococcus pyogenes* 7008	nested PCR	*tet (O)*		[8]
Small crickets	*Acheta domesticus*	Netherlands Market	*Lactobacillus casei/paracasei* ILC2279	PCR	*tet (M)*		[8]
Locusts	*Locusta migratoria*	Netherlands Market	*Staphylococcus aureus* COL.	PCR	*tet(K)*		[8]
Mealworm larvae	*Tenebrio molitor*	Netherlands Market	*Staphylococcus aureus* COL.; *Enterococcus italicus* 1102; *Staphylococcus aureus* COL.	PCR	*tet(M), tet(S), tet(K)*		[8]
Giant waterbugs	*Belostomatidae*	Thailand Market	*Enterococcus hirae* Api 2.16; *Staphylococcus aureus* COL.; *Staphylococcus aureus* ATCC 2921	nested PCR	*erm(B); tet(K); blaZ*		[8]
Black ants	*Polyrhachis*	Thailand Market	*Staphylococcus aureus* COL.	PCR	*tet(K)*		[8]
Black ants	*Polyrhachis*	Thailand Market	*Staphylococcus aureus* COL.	nested PCR	*erm(B); erm(C); blaZ*		[8]
Winged termite alates	*Termitoidae*	Thailand Market	*Enterococcus hirae* Api 2.16; *Staphylococcus spp.* SE12; *Enterococcus italicus* 1102; *Staphylococcus aureus* COL.; *Staphylococcus aureus* ATCC 2921	nested PCR	*erm(B); erm(C); tet(S), tet(K); blaZ*		[8]
Rhino beetles	*Hyboschema contractum*	Thailand Market	*Enterococcus hirae* Api 2.16; *Enterococcus italicus* 1102; *Staphylococcus aureus* COL.; *Staphylococcus aureus* ATCC 2921	nested PCR	*erm(B); tet(S), tet(K); blaZ*		[8]
Mole crickets	*Gryllotalpidae*	Thailand Market	*Enterococcus italicus* 1102; *Staphylococcus aureus* COL.	PCR	*erm(B); tet(S), tet(K)*		[8]
Mole crickets	*Gryllotalpidae*	Thailand Market	*Staphylococcus aureus* ATCC 2921	nested PCR	*blaZ*		[8]
Silkworm pupae	*Bombyx mori*	Thailand Market	*Enterococcus italicus* 1102; *Staphylococcus aureus* COL.; *Staphylococcus aureus* ATCC 2921	nested PCR	*tet(S), tet(K); blaZ*		[8]
Black scorpions	*Heterometrus longimanus*	Thailand Market	*Enterococcus italicus* 1102; *Staphylococcus aureus* COL.	PCR	*tet(S), tet(K)*		[8]
Giant Line Green Stick insect	*Diaphroedes gigantea*		*Sphingobacterium multivorum*	broth microdilution assay		Tetracycline	[14]
Giant Line Green Stick insect	*Diaphroedes gigantea*		*Microbacterium oxydans*,	broth microdilution assay		Ciprofloxacin; Tetracycline	[14]
Giant Line Green Stick insect	*Diaphroedes gigantea*		*Bacillus amyloliquefaciens*	broth microdilution assay		Ciprofloxacin; Tetracycline	[14]
Diamondback moth	*Plutella xylostella*		*Sanguibacter keddieii*	broth microdilution assay		Ampicillin; Chloramphenicol; Ciprofloxacin; Kanamycin	[14]
Diamondback moth	*Plutella xylostella*		*Raoultella terrigena*	broth microdilution assay		Ampicillin; Chloramphenicol; Kanamycin; Rifampicin	[14]
Cinnabar moth	*Tyria jacobeae*		*Bacillus licheniformis*	broth microdilution assay		Ampicillin; Chloramphenicol; Tetracycline	[14]
Cinnabar moth	*Tyria jacobeae*		*Staphylococcus epidermidis*	broth microdilution assay		Ampicillin; Kanamycin	[14]
Cinnabar moth	*Tyria jacobeae*		*Staphylococcus warneri*	broth microdilution assay		Ciprofloxacin; Rifampicin; Tetracycline	[14]
Cinnabar moth	*Tyria jacobeae*		*Burkholderia fungorum*	broth microdilution assay		Ciprofloxacin; Kanamycin	[14]
Death’s-head Hawkmoth	*Acherontia atropos*		*Enterobacter asburiae*	broth microdilution assay		Ampicilin; Chloramphenicol; Ciprofloxacin; Kanamycin; Rifamplcin; Tetracycline	[14]
Death’s-head Hawkmoth	*Acherontia atropos*		*Pseudomonas putida*	broth microdilution assay		Chloramphenicol; Kanamycin; Rifampicin; Tetracycline	[14]
Death’s-head Hawkmoth	*Acherontia atropos*		*Roultella terrigena*	broth microdilution assay		Ampicillin; Chloramphenicol; Kanamycin	[14]
Beet Armyworm	*Spodoptera exigua*		*Microbacterium paraoxydans*	broth microdilution assay		Ampicillin; Ciprofloxacin	[14]
Beet Armyworm	*Spodoptera exigua*		*Bacillus aquimaris*	broth microdilution assay		Rifampicin; Chloramphenicol; Kanamycin	
Beet Armyworm	*Spodoptera exigua*		*Bacillus vietnamensis*	broth microdilution assay		Ampicillin; Chloramphenicol; Ciprofloxacin; Kanamycin; Rifampicin	[14]
Beet Armyworm	*Spodoptera exigua*		*Rhizobium pusense*	broth microdilution assay		Ciprofloxacin; Kanamycin; Tetracycline	[14]
Rosemary beetle	*Chrysolina americana*		*Staphylococcus epidermidis*	broth microdilution assay		Ampicillin; Ciprofloxacin; Kanamycin	[14]
Mealworm larvae	*Tenebrio molitor* L.		Ampicillin-resistant lactic acid bacteria	pour plating		Ampicillin	[15]
Mealworm larvae	*Tenebrio molitor* L.		Vancomycin-resistant lactic acid bacteria	pour plating		Vancomycin	[15]
Mealworm larvae	*Tenebrio molitor* L.		Ampicillin-resistant *enterococci*	speard plating		Ampicillin	[15]
Mealworm larvae	*Tenebrio molitor* L.		HLAR *enterococci*	spread plating		Gentamicin	[15]
Mealworm larvae	*Tenebrio molitor* L.		Vancomycin-resistant *enterococci*	spread plating		Vancomycin	[15]
Mealworm larvae	*Tenebrio molitor* L.		Gentamicin-resistant *staphylococci*	spread plating		Gentamicin	[15]
Mealworm larvae	*Tenebrio molitor* L.		Erythromycin-resistant *staphylococci*	spread plating		Erythromycin	[15]
Mealworm larvae	*Tenebrio molitor* L.		Tetracycline-resistant *staphylococci*	spread plating		Tetracycline	[15]
Mealworm larvae	*Tenebrio molitor* L.		Vancomycin-resistant coagulase-positive *staphylococci*	spread plating		Vancomycin	[15]
Mealworm larvae	*Tenebrio molitor* L.		Ampicillin-resistant *Enterobacteriaceae*	pour plating		Ampicillin	[15]
Mealworm larvae	*Tenebrio molitor* L.		Gentamicin-resistant *Enterobacteriaceae*	pour plating		Gentamicin	[15]
Mealworm larvae	*Tenebrio molitor* L.		Gentamicin-resistant *Pseudomonadaceae*	pour plating		Gentamicin	[15]
Mealworm larvae	*Tenebrio molitor* L.		*Enterococcus italicus*	PCR	*tet(S)*		[15]
Mealworm larvae	*Tenebrio molitor* L.		*Enterococcus hirae* Api 2.16; *Lactobacillus casei/paracasei* ILC2279; *Staphylococcus aureus* COL	N-pcr	*erm(B); tet(M); tet (K)*		[15]
Crickets	*Acheta domesticus*	Austria, Belgium, France, and the Netherlands	Lactic acid bacteria; *Enterobacteriaceae; Pseudomonadaceae; Listeria monocytogenes*	PCR	*erm(B); erm(C); tet(S), tet(K); tet(S), tet(K); blaZ; aac-aph*		[16]
Mealworms	*Tenebrio molitor* L.	Belgium	Total mesophilic aerobes; spore-forming bacteria; lactic acid bacteria; *Enterobacteriaceae*	PCR; N-pcr	*erm(B); vanA; tet(M); tet(S); tet(K); aac-aph*		[16]
Mealworms	*Tenebrio molitor* L.	Netherlands	Total mesophilic aerobes; spore-forming bacteria; lactic acid bacteria; *Enterobacteriaceae*	PCR; N-pcr	*erm(B); erm(C); tet(M); tet(S); tet(K); mecA; aac-aph*		[16]
Mealworms	*Tenebrio molitor* L.	Thailand	Total mesophilic aerobes; spore-forming bacteria; lactic acid bacteria; *Enterobacteriaceae*	PCR; N-pcr	*erm(B); erm (C); tet(O); tet(S); tet(K); mecA; aac-aph*		[17]
Mealworms	*Tenebrio molitor* L.	France	Total mesophilic aerobes; spore-forming bacteria; lactic acid bacteria; *Enterobacteriaceae*	PCR; N-pcr	*erm(A); erm(B); erm©; vanA; vanB; tet(M); tet(O); tet(S); tet(K); mecA; blaZ; aac-aph*		[17]
House cricket	*Acheta domesticus*	Europe	total aerobic mesophilic microbiota, *lactobacilli, Enterobacteriaceae,* total coliforms, *staphylococci,* and *enterococci*	PCR; N-pcr	*tetM*		[18]
Mealworms	*Tenebrio molitor* L.	Belgium, the Netherlands, Thailand	*Staphylococcus* sp., *Bacillus* sp., *Weissella* sp., *Eikenella* sp., *Exiguobacterium* sp.	PCR; N-pcr	*bla(OXA−48); bla(NDM−1); tet(M), tet(K), tet(S); erm(B), erm(C); aac-aph*		[19]
Grasshopper	*Locusta migratoria migratorioides*	Thailand	*Staphylococcus* sp., *Bacillus* sp., *Weissella* sp., *Eikenella* sp., *Exiguobacterium* sp.	PCR; N-pcr	*blaVIM*		[19]
Grasshopper	*Locusta migratoria migratorioides*	Belgium, the Netherlands, Thailand	*Staphylococcus* sp., *Bacillus* sp., *Weissella* sp., *Eikenella* sp., *Exiguobacterium* sp.	PCR; N-pcr	*bla(OXA−48); bla(NDM−1); tet(M), tet(K), tet(S); erm(B), erm(C); aac-aph; blaZ*		[19]
Crickets	*Acheta domesticus*	Belgium and the Netherlands		PCR; N-pcr	*tet(O), tet(K), tet (M), tet(S), erm(B)*		[20]

## Data Availability

The original contributions presented in the study are included in the article, further inquiries can be directed to the corresponding author.
